# Engaging Parents in Analgesia Selection and Racial/Ethnic Differences in Analgesia Given to Pediatric Patients Undergoing Urologic Surgery

**DOI:** 10.3390/children7120277

**Published:** 2020-12-07

**Authors:** Carl Lo, Patrick A. Ross, Sang Le, Eugene Kim, Matthew Keefer, Alvina Rosales

**Affiliations:** 1 Department of Anesthesiology Critical Care Medicine, Division of Pain Medicine, Children’s Hospital Los Angeles, Los Angeles, CA 90027, USA; paross@chla.usc.edu (P.A.R.); sale@chla.usc.edu (S.L.); eugekim@chla.usc.edu (E.K.); alrosales@chla.usc.edu (A.R.); 2 Keck School of Medicine, University of Southern California, Los Angeles, CA 90033, USA; mkeefer@chla.usc.edu; 3 Department of Pediatrics, Children’s Hospital Los Angeles, Los Angeles, CA 90027, USA

**Keywords:** health disparities, regional anesthesia, caudal regional block, pediatric pain management, race, ethnicity

## Abstract

Background: Family-centered care aims to consider family preferences and values in care delivery. Our study examines parent decisions regarding anesthesia type (caudal regional block or local anesthesia) among a diverse sample of children undergoing urologic surgeries. Differences in anesthesia type were examined by known predictors of health disparities, including child race/ethnicity, parental English proficiency, and a proxy for household income. Methods: A retrospective review of 4739 patients (including 25.4% non-Latino/a White, 8.7% non- Latino/a Asians, 7.3% non-Latino/a Black, 23.1% Latino/a, and 35.4% others) undergoing urologic surgeries from 2016 to 2020 using univariate and logistic regression analyses. Results: 62.1% of Latino/a parents and 60.8% of non-Latino/a Black parents did not agree to a regional block. 65.1% of Spanish-speaking parents with limited English Proficiency did not agree to a regional block. Of parents from households below poverty lines, 61.7% did not agree to a caudal regional block. In regression analysis, Latino/a and non- Latino/a Black youth were less likely to receive caudal regional block than non- Latino/a White patients. Conclusions: We found disparities in the use of pediatric pain management techniques. Understanding mechanisms underlying Latino/a and non- Latino/a Black parental preferences may help providers reduce these disparities.

## 1. Introduction

Family-centered pain management emphasizes the importance of engaging parents in decision making regarding their child’s pain management [1]. Family- and patient- centered approaches are thought to bridge demographic and social differences between providers and patients to better address and prevent health disparities [2]. In perioperative settings, family-centered practices often include parent involvement in decisions regarding types of perioperative pain management. Parents may be asked their preferences regarding regional anesthesia approaches (e.g., local infiltration or caudal regional block) or systemic opioid use. However, there is limited knowledge of parental preferences as they relate to pain management during elective procedures, and whether parent-provider decisions vary systematically. Given documented health disparities in pain management [3], it is important to examine parent preferences and whether these vary by race, ethnicity, household income, and salient cultural factors such as English language proficiency.

The ability to examine perioperative health equity depends on existing evidence, and how clearly it identifies gold standard approaches to peri-operative pain management. Several analgesia techniques exist for patients undergoing urologic procedures, including caudal regional block, local anesthetic infiltration, systemic opioids, and topical local anesthetic. A Cochrane review of efficacy of caudal regional blocks compared to other techniques on children found less need for rescue analgesia, and less nausea and emesis for patients receiving caudal regional blocks versus systemic opioids without any regional anesthesia [4]. Serious complications such as hemodynamic instability, arrhythmia, seizures, and respiratory depression are considered unusual [5], and long-term complications such as nerve damage or paralysis are extremely rare [6]. However, study findings have also been mixed. A systematic review of caudal regional blocks for inguinal hernia repair in children did not yield significant differences in postoperative pain scores or need for rescue analgesia compared with surgical wound infiltration [5]. While a single gold standard approach does not exist and the literature is not definitive, caudal regional blocks seem to improve perioperative pain control; reduce potential for side effects [7]; and enable earlier recovery, ambulation, wakefulness, and faster discharge times from postoperative recovery units [8]. Given the evidence base, at our institution it is standard practice to offer a caudal regional block to all patients six years and younger having urologic procedures below the umbilicus. Regardless of demographic differences, it is imperative to examine whether systematic differences exist in youth who end up receiving blocks.

Racial and ethnic disparities in perioperative pain management have been extensively documented, though mostly in adult studies. Adult studies demonstrate that ethnicity, race, and insurance status predict the types of anesthetic patients receive, and indicate inadequate pain management in minority groups [9,10]. Latino/a and Black patients are also less likely than non- Latino/a White patients to receive epidural analgesia [11]. A large retrospective cohort study on 81,883 pregnant women between 1998 and 2003 demonstrated that patients with private insurance are more likely to receive labor epidurals than those without, though the role of patient decision-making was uncertain [11]. Compared to adult studies, there is relatively limited research examining perioperative pediatric pain management. Latino/a parents were found to report more fear and avoidance regarding analgesic use for their children, and provide fewer than the recommended number of doses of analgesic to their children on the first day after outpatient surgery [12]. Relatedly, language and low English proficiency were found to impact health outcomes, even with interpretation services available [13].

The primary aim of this paper is to elucidate if child race and ethnicity predict the likelihood of receiving a caudal regional block for children undergoing urologic surgeries. Secondary aims are to examine whether type of analgesia technique varies in households with income below federal poverty levels or based on parents’ limited English proficiency (LEP). We hypothesized that parents of children identified as Latino/a and non- Latino/a Black ethnicity/race, living in a household with income below poverty level, and with LEP would be less likely to consent for their children to receive a caudal regional block.

## 2. Materials and Methods

The present study is a single-site retrospective study of pediatric patients whose parents were offered a caudal regional block for urologic surgery at an urban, academic, tertiary children’s hospital. We obtained local institutional review board (IRB) approval (CHLA-19-00457). By consensus agreement between the Pain Medicine Team and the Division of Urologic Surgery at CHLA, all children six years or younger are offered caudal regional blocks if the surgical procedure is appropriate. We use this age cutoff in older children because the sacral canal anatomy varies greatly, and local anesthetics spread is unpredictable. Our Pain Medicine team offers caudal regional blocks on the day of surgery to parents verbally, covering standard benefits and risks. For children older than six years, use of caudal regional block is at the discretion of the Pain Medicine Team. Therefore, data were extracted from the electronic anesthesia record of all children 6 years of age or less undergoing urologic surgery from January 2016 to August 2020. The surgical diagnosis and procedure information was reviewed independently by the authors for every patient. Cases were excluded if they did not meet criteria to be offered caudal regional block. For example, in rare cases when other types of regional anesthetics such as epidurals or peripheral nerve blocks were used these cases were excluded. (Supplemental Table S1).

Patient demographic data were collected, including child race (non- Latino/a White, non-Latino/a Black, and non-Latino/a Asian, hereafter referred to as White, Latino/a, Black, and Asian) as stated by the parents at the time of admission, ethnicity (Latino/a) as stated by the parents, gender, age, parent English proficiency (defined as preferring to communicate in English or other primary non-English language), and patient insurance type (as proxy for household income). While there was overlap between Latino/a ethnicity and race, the overlap was <1% for Latino/a Black and Latino/a Asian and was excluded from analysis. Therefore, the groups Latino/a, White, Asian, and Black were used based on limitations of available data and proportions in the sample size. Insurance type (private versus government insurance) was used as a proxy for household income (above or below poverty level respectively). For parents with LEP or who preferred their primary language, live in-person interpretation was provided in the perioperative setting. The primary outcome measure of the study was parent agreement/disagreement for the patient to receive caudal regional block during the surgical procedure.

### Statistical Analysis

We performed statistical analysis using Stata version 15 (Stata Corp, College Station, TX, USA). Descriptive statistics were completed for patient demographic information. Median and 1st, 3rd Interquartile ranges were used for continuous data as it was not normally distributed. Frequency and percentage were used for categorical data. Effect size for secondary outcome variables was calculated using Cohen’s d for continuous variables and Cramer’s V for categorical variables. Subgroup analysis was performed for Latino/a families evaluating use of caudal regional block based upon stated preferred language, and difference was evaluated using Pearson’s *χ*^2^. Univariate analysis for the outcome of private versus government insurance was performed with expected versus observed *χ*^2^. Univariate analysis against the secondary outcome insurance type was performed with expected versus observed *χ*^2^.

Multivariable analysis on the outcome use of caudal regional block was performed using a logistic regression model. Model selection was based upon univariate outcome and primary hypothesis. Gender, age, weight, and height were examined as potential modulating factors and not included due to small effect size (Table 1).

## 3. Results

A total of 4739 patients’ data met all inclusion and no exclusion criteria and were used for our analysis (Table 1). The group that received caudal regional block were younger and smaller than those that did not. There was a small difference in the percent male patients in each group that was statistically significant but not clinically relevant Table 2.

Parent preferred language was available for 4399 patients (Table 1). There was a statistically significant difference in the distribution of primary language between patient’s families that agreed to caudal regional block. Of those receiving caudal regional block, there was a higher percentage of primary English speakers and a lower percentage of primary Spanish speakers. Subgroup analysis of only the Latino/a families and language was shown in Table 2. Of the Latino/a families that declared Spanish as their primary language (410), 34.9% agreed to caudal regional block and 65.1% did not. 

Race and ethnicity were available for all 4739 patients (Table 1). There was a statistically significant difference in the distribution of race/ethnicity between patient’s parents that agreed to caudal regional block. Of those receiving caudal regional block, there was a higher percentage of White or Asian patients. White and Asian families were more likely to choose caudal regional block than Latino/a families. Of the Latino/a families, (1096) 37.9% of parents agreed to caudal regional block and 62.1% did not.

Household income (insurance type proxy) was available for 4685 patients (Table 1). There was a statistically significant difference between groups, with caudal regional block being accepted by a higher percentage of families with private insurance and a lower percentage with government insurance. Of the families with government insurance, (2820) 38.3% agreed to caudal regional block and 61.7% did not.

For the outcome household income, English language proficiency was available for 4345 patients (Table 3). There was a statistically significant difference in the distribution of primary language between patient’s families that had private insurance. Of those with private insurance, there was a higher percentage of primary English speakers and a lower percentage of primary Spanish speakers. Of the families that declared Spanish as their primary language (725), 3% had private insurance and 97% had government insurance.

For the outcome, insurance type and race/ethnicity were available for all 4685 patients (Table 3). There was a statistically significant difference in the distribution of race/ethnicity between patient’s families that had private insurance. Of those with private insurance, there was a higher percentage of families that declared themselves to be White and Asian. Of those with private insurance, there was a lower percentage of families who declared themselves to be Latino/a. Of the families that identified their child as Latino/a, (1081) 12.6% had private insurance and 87.4% had government insurance.

The logistic regression model with the primary outcome of use of caudal regional block is shown in Table 4. Overall, there is a statistically significant lower odds ratio for the use of caudal regional block for families who declare as Latino/a and Black than the baseline White. For Latino/a, the odds ratio was 0.72 ((95% CI (confidence interval) 0.61–0.85)). Demographic data, language, and insurance type were present in the development of the model. Primary language was not statistically significant in the logistic regression. Use of government insurance was associated with a statistically significant lower odds ratio for the use of caudal regional block than the baseline private insurance.

## 4. Discussion

The present study highlights the importance of examining pediatric perioperative pain management practices by race and ethnicity. Primary findings indicate that race and ethnicity play significant roles in the likelihood that children receive caudal regional blocks for urological procedures. Latino/a and Black youth are less likely to receive a caudal regional block than White youth. Latino/a and Black groups were also more likely to have household incomes under poverty level (government-assisted insurance). The present study is the first to explore association between ethnicity, English language proficiency, and household income with the likelihood for parents consenting to caudal regional block in pediatric urologic surgeries. This study adds to the current literature describing pain management disparities disproportionately impacting Latino/a and Black patients [12,14,15]. 

A strong correlation was established between race/ethnicity and household income (insurance type). In our study, about 67.7% of White patients have private insurance, and the vast majority of our CHLA Latino/a patients have government insurance at 87.4%. Studies examining insurance type as a proxy for family income have also found similar trends where insurance type can influence clinical treatments in adult outpatient ambulatory inguinal hernia surgery [9]. Other secondary aims of this study are to examine English proficiency (request for interpretation services as a proxy) as a predictor of obtaining caudal regional block consent. Language accessibility has been an often-cited reason LEP patients reject medical treatment [13]. It is possible that consistent use of interpretation services for parents with LEP is an effective intervention. Additionally, a resourceful interpretation service, rather than a simple word-by-word translation, may be an essential link to better convey equal healthcare opportunities. Increasing diversity of clinicians to better represent lived experiences, culture, and language of diverse patient populations may enhance the effectiveness of parent-provider discussions and lead to more optimal analgesia selection for pediatric patients.

The reasons underlying parents’ decisions on perioperative pain management may be multifactorial and are beyond the scope of this study. Studies examining microsystem factors related to perioperative pain management have found family factors can play a significant role. Rosales et al. found that Latino/a parents felt anxious before their child’s surgery [16], felt significantly concerned about the secondary effects of analgesics, and gave their children less than recommended doses of pain medication following surgery, leading to significant patient postoperative pain [12]. Clinician-related factors include communication approaches and implicit race and social class bias that may impact care [17]. For example, providers may assume similar ways of communicating anesthesia options to be effective among different parent groups.

From a broader system viewpoint, assumptions of Latino/a and Black parent decisions to deny caudal regional blocks may point to “cultural factors” as an explanation. However, rather than reflecting cultural values, beliefs or practices, we contend that preferences in surgical analgesia are more likely protective responses and rooted in multi-generational histories of health, social inequities, and disparities (e.g., exposure to racism) [18]. Latino/a and Black parents may elect more conservative approaches due to limited provider trust, or insufficient time to develop trust and understanding of a caudal regional block. Fear or mistrust of healthcare systems has also been implicated in the underuse of epidural analgesia among ethnic minority parents. As much as 36% have been found to discourage their family and friends getting an epidural [19]. Clinicians should understand the possibilities of these histories and inherited vulnerability [20] to health inequities in an acute care surgical setting. Future clinical considerations include examining and identifying possible areas for improvement in the process of consent. For example, providers may give parents multiple opportunities to learn about analgesic options before the day of surgery.

There are limitations to this study. The information presented to the families regarding regional caudal block was done in a clinical setting. We are unable to account for the time spent by each practitioner in obtaining consent. We cannot assure that the information was presented in the same manner. The determination of race/ethnicity was made by the families of these patients to reflect their child. Our system did not have more granular information to reflect multi-racial families. We are also not able to determine if the race/ethnicity declared for the child is the same as their parents’.

Because this is a cross-sectional retrospective study, it is difficult to determine why there is such a significant disparity in the likelihood to consent for caudal regional blocks in different racial and ethnic groups. It is also difficult for our study to delineate the variables of ethnicity, language, and insurance type separately. These variables have significant overlap and are closely intertwined statistically and theoretically. Future studies are needed to better assess specific mechanisms underlying parent decision-making. Triangulating methods to include qualitative and community-based approaches where community collaborators are involved in data interpretation may be particularly beneficial [21]. It is important to clarify that the literature does not have a clear indication that the use of caudal regional block vs. local anesthetic is considered a gold standard. This prevents us from definitively concluding there is health inequity. However, the cost of waiting to monitor pain management trends until gold standard approaches are determined may result in long periods of time where racial and ethnic minority groups receive sub-optimal care.

## 5. Conclusions

Latino/a and Black pediatric patients are less likely to receive caudal regional blocks than White peers for urologic procedures. Family-centered approaches may benefit from considering the role of race and ethnicity in decision-making. It is important that clinicians and health institutions practice ongoing vigilance of clinically significant differences in pain management approaches based on race and ethnicity. This mindset should be integrated into standard practices for pediatric hospitals as part of a family-centered philosophy that is centered in family experiences in social and healthcare systems.

## Figures and Tables

**Table 1:** Differences between caudal regional block and no caudal regional block. Data is for each individual surgery and the patient characteristics at the time of surgery.

	Regional	No Regional	Effect Size	Effect Size 95% CI
	*n* = 1931	*n* = 2808		
Gender (Male)	1803 (93.4%)	2704 (96.3%)	−0.067	−0.096 to −0.038
Age (Year)	1.49 (0.89, 2.87)	1.88 (0.94, 3.71)	0.230	0.172 to 0.288
Weight kg	11.1 (9.0, 14.6)	12.4 (9.6 16.4)	0.248	0.190 to 0.306
Height cm	80 (71, 94)	85 (74, 101)	0.217	0.159 to 0.276
Language				
English	1507 (83.1%)	2066 (79.9%)		
Spanish	267 (14.7%)	466 (18.0%)		
Other	39 (2.2%)	54 (2.1%)		
Race/Ethnicity				
Latino/a	415 (21.5%)	681 (24.3%)		
White	574 (29.7%)	631 (22.5%)		
Black	136 (7.0%)	211 (7.5%)		
Asian	193 (10.0%)	219 (7.8%)		
Other	613 (31.8%)	1066 (40.0%)		
Household Income (Insurance Type)				
Private	833 (43.5%)	1032 (37.2%)		
Government	1080 (56.5%)	1740 (62.8%)		

Data is presented as number (percentage) or median (1, 3 interquartile range). Measure of effect size is presented for continuous variables as Cohen’s d and 95% CI and for categorical variables as Cramer’s V and 95% CI. CI: Confidence Interval.

**Table 2:** Subgroup analysis, and use of regional anesthesia for Latino/a families by primary language.

	Regional	No Regional	*p* Value
English	260 (39.6%)	396 (60.4%)	0.119
Spanish	143 (34.9%)	267 (65.1%)	

Data are presented as number (percentage) and difference between groups calculated using Chi square.

**Table 3:** Private versus government insurance type versus primary language and race/ethnicity.

	Private Insurance	Government Insurance	
Language	*n* = 1670	*n* = 2675	
English	1636 (98.0%)	1891 (70.7%)	<0.001
Spanish	22 (1.3%)	703 (26.3%)	
Other	12 (0.7%)	81 (3.0%)	
Race/Ethnicity	*n* = 1865	*n* = 2820	
Latino/a	136 (7.3%)	945 (33.5%)	<0.001
White	811 (43.5%)	387 (13.7%)	
Black	78 (4.2%)	265 (9.4%)	
Asian	230 (12.3%)	179 (6.4%)	
Other	610 (32.7%)	1044 (37.0%)	

Data is presented as number (percentage). Statistical differences determined with expected/observed Chi square.

**Table 4:** Logistic regression for the primary outcome use of caudal regional block.

Variable	Odds Ratio	95% CI	*p* Value
White	Baseline	Baseline	Baseline
Latino/a	0.72	0.61–0.85	<0.001
Black	0.73	0.57–0.93	0.012
Asian	0.97	0.78–1.22	0.812
Other	0.65	0.56–0.75	<0.001
English	Baseline	Baseline	Baseline
Spanish	0.93	0.77–1.12	0.436
Other	0.93	0.60–1.45	0.746
Private Insurance	Baseline	Baseline	Baseline
Government Insurance	0.85	0.74–0.98	0.028

## Supplementary Materials

The following are available online at https://www.mdpi.com/2227-9067/7/12/277/s1, Supplemental Table S1: Procedures appropriate for caudal regional block and ones not offered.
