# Supplementary material for: Engaging Parents in Analgesia Selection and Racial/Ethnic Differences in Analgesia Given to Pediatric Patients Undergoing Urologic Surgery
Source: Children (Basel). 2020 Dec 7;7(12):277. doi: 10.3390/children7120277 (PMC7762314; doi:10.3390/children7120277)
Supplement: Supplementary file 1 [file children-07-00277-s001.pdf]

Supplementary Materials:

Supplemental Table #1. Procedures appropriate for caudal regional block and ones not offered.

Appropriate for Regional Anesthesia	Not offered Regional Anesthesia
Abdominal exploration diagnostic	Cystoscopy
Bladder neck reconstruction	Cystoscopy with additional procedures (stent)
Chordee correction	Labial lysis of adhesions
Circumcision	Percutaneous laser nephrolithotomy
Epispadias repair	Ureteral stent placement
Genitoplasty feminizing	Ureteroscopic stone removal
Hernia inguinal repair	Vaginoscopy
Hernia umbilical repair	Wound irrigation and debridement
Hydrocelectomy	
Hypospadias repair	
Inguinal exploration for undescended testes	
Meatoplasty	
Nephrectomy	
Nephrouretectomy	
Orchiectomy	
Orchiopexy	
Ovarian cystectomy	
Penile lesion excision	
Penis buried / hidden release	
Pyeloplasty	
Scrotoplasty	
Testicular mass excision	
Ureteric re-implantation	
Ureterocutaneous fistula closure	
Urethroplasty	
Vaginoplasty	
Vesicostomy	
